# Plasma Levels of Soluble HLA-E and HLA-F at Diagnosis May Predict Overall Survival of Neuroblastoma Patients

**DOI:** 10.1155/2013/956878

**Published:** 2013-11-21

**Authors:** Fabio Morandi, Giuliana Cangemi, Sebastiano Barco, Loredana Amoroso, Maria Giuliano, Anna Rita Gigliotti, Vito Pistoia, Maria Valeria Corrias

**Affiliations:** ^1^Laboratorio di Oncologia, Istituto Giannina Gaslini, Via G. Gaslini 5, 16148 Genova, Italy; ^2^Laboratorio di Analisi, Istituto Giannina Gaslini, Via G. Gaslini 5, 16148 Genova, Italy; ^3^Oncologia Clinica, Istituto Giannina Gaslini, Via G. Gaslini 5, 16148 Genova, Italy; ^4^Oncoematologia, Ospedale Pausillipon, Via Mario Fiore 6, 80123 Napoli, Italy; ^5^Epidemiologia, Biostatistica e Comitati, Istituto Giannina Gaslini, Via G. Gaslini 5, 16148 Genova, Italy

## Abstract

The purpose of this study was to identify the plasma/serum biomarkers that are able to predict overall survival (OS) of neuroblastoma (NB) patients. Concentration of soluble (s) biomarkers was evaluated in plasma (sHLA-E, sHLA-F, chromogranin, and B7H3) or serum (calprotectin) samples from NB patients or healthy children. The levels of biomarkers that were significantly higher in NB patients were then analyzed considering localized or metastatic subsets. Finally, biomarkers that were significantly different in these two subsets were correlated with patient's outcome. With the exception of B7H3, levels of all molecules were significantly higher in NB patients than those in controls. However, only chromogranin, sHLA-E, and sHLA-F levels were different between patients with metastatic and localized tumors. sHLA-E and -F levels correlated with each other but not chromogranin. Chromogranin levels correlated with different event-free survival (EFS), whereas sHLA-E and -F levels also correlated with different OS. Association with OS was also detected considering only patients with metastatic disease. In conclusion, low levels of sHLA-E and -F significantly associated with worse EFS/OS in the whole cohort of NB patients and in patients with metastatic NB. Thus, these molecules deserve to be tested in prospective studies to evaluate their predictive power for high-risk NB patients.

## 1. Introduction

 Neuroblastoma (NB) is a neuroectodermal tumor originating from the sympathetic nervous system and represents the most common extracranial solid malignancy in childhood [1]. According to the new NB risk classification [2], the variables currently employed for risk stratification are age at diagnosis (< or ≥18 months), stage (localized disease, without or with image-defined risk factors [3], and metastatic disease), and genetic abnormalities (*MYCN* amplification and 11q deletion). Thus, risk stratification of patients to different therapeutic regimens does not include soluble biomarkers, although a soluble biomarker, able to predict overall survival (OS) of patients with NB, would allow to reduce invasive procedures before starting the treatments.

In the last years, we have demonstrated that different molecules such as soluble (s)HLA-G, ICAM-1, cytoplasmic melanoma-associated antigen (CYT-MAA), and high-molecular weight melanoma associated antigen (HMW-MAA) were more abundant in NB patients' plasma than in healthy children [4, 5]. Moreover, high serum levels of sHLA-G and CYT-MAA at diagnosis predicted a worse event-free survival (EFS), thus suggesting a potential use of these molecules as surrogate biomarkers [4, 5]. We have also demonstrated that high levels of DKK-1 associate with poor clinical response [6] and high VMA/HVA ratio associated with a better EFS, but only in patients with localized *MYCN* nonamplified tumors [7]. Thus, no soluble biomarker able to predict overall survival (OS) of NB patients is currently available.

Here, we have tested different soluble plasma/serum molecules, precisely: (i) sHLA-E and sHLA-F, two molecules belonging to HLA-class Ib family as sHLA-G, that was previously characterized in NB [4], (ii) B7H3, a glycoprotein belonging to the costimulatory B7 family, whose expression in NB tumors associated with worse EFS [8]; (iii) calprotectin, a calcium-binding protein of the S100 family, recently identified as specific marker for bone marrow-infiltrating NB cells [9], and (iv) chromogranin, characterized as surrogate biomarker for neuroendocrine tumors [10, 11]. The levels of these molecules were first compared between healthy children and NB patients. Then, levels of molecules significantly higher in NB patients were correlated to stage, age, and clinical outcome.

## 2. Materials and Methods

### 2.1. Patients and Controls

Plasma samples were collected at diagnosis from 84 consecutive NB patients admitted between 1995 and 2012 at the Oncology centers belonging to the Associazione Italiana di Emato Oncologia Pediatrica. Samples were immediately centralized at the reference laboratory in Gaslini Institute. 

NB patients were staged according to the International Neuroblastoma Staging System [12]. However, for some of the analyses, they were allocated to the different risk groups according to the INRG [2]. After diagnosis of NB, the study patients were treated according to the Italian Neuroblastoma protocols [13] until the commencement of the various International Society of Pediatric Oncology European Neuroblastoma Group (SIOPEN) protocols [14–17]. All protocols were approved by the local Institutional Review Boards and a written consent allowing the collection of samples and the use of clinical and nongenetic data for clinical research was signed by the patients or their guardians.

The demographic, clinical, and followup data (at January 31th, 2013) of the 84 study patients (7 stage 1, 8 stage 2, 22 stage 3, 42 stage 4, and 5 stage 4S) were retrieved from the Italian NB Registry [13]. NB patients' characteristics and clinical features at diagnosis and follow-up are summarized in Table 1. The study patients were representative of the entire NB population registered in the same time period. The median followup was 54 months (range: 1.06–156.3 months).

Serum samples necessary to perform calprotectin analysis were collected at diagnosis from 33 NB patients admitted at the Gaslini Institute between January 2010 and December 2012. 

As controls, 75 plasma and 34 serum samples collected between January 2010 and December 2012 from children admitted at the Gaslini Institute for accidental injuries were used. The study was approved by the Ethics Committee of the G. Gaslini Institute, Genoa, Italy.

### 2.2. ELISA

Enzyme-linked immunosorbent assay (ELISA) for sHLA-E was performed as previously described [18]. Results are expressed as arbitrary units/mL (1 unit = quantity of sHLA-E or -F in 1 *μ*g of total extract). The same experimental protocol was used for sHLA-F and sB7H3, using capture reagent rabbit polyclonal anti-HLA-F antibody (Abgent, San Diego, CA, USA) or mouse monoclonal 5B14 antibody (kindly donated by Dr. Cristina Bottino), respectively. As detection reagent, HRP-conjugated anti-*β*2 microglobulin mAb (Exbio) or HRP-conjugated mouse monoclonal 376.96 antibody (kindly donated by Dr. Soldano Ferrone) was used, respectively. 376.96 antibody was conjugated with HRP using HRP conjugation kit (Thermo Fisher Scientific Inc., Rockford, IL, USA) following the manufacturer's protocol.

Chromogranin specific ELISA (Chromogranin NEOLISA, Euro-DiagnosticaAB, Malmö, Sweden) was carried out using 100 *μ*L of plasma, whereas calprotectin specific ELISA (Calprotectin S100 A8/A9 Elisa Kit, Immundiagnostik, Bensheim, Germany) was carried out using 100 *μ*L of serum. Both kinds of ELISA were performed following the manufacturer's protocol. Specific absorbance at 450 nm, normalized to absorbance at 620 nm, was measured in a DSX spectrophotometer (Dynex Technologies, West Sussex, UK). Results are expressed as pg/mL and ng/mL, respectively.

### 2.3. Statistics

Normal distribution of data was tested using Kolmogorov-Smirnov test, using Prism software (GraphPad Software Inc., La Jolla, CA). The plasma/serum levels measured in patients and controls were compared by Student's *t*-test or Mann-Whitney *U* test, depending on data distribution, using Prism software.

Correlations between plasma levels of different antigens were calculated by Spearman's test, and correlations between levels of plasma molecules and other predictive parameters were calculated by Fisher exact test, using Prism software. 

To determine the cut-off level of each antigen to be considered elevated, ROC curves were constructed using MedCalc software (Mariakerke, Belgium). Cut-off values were considering the following variables: (i) plasma levels of chromogranin, HLA-E, or HLA-F; (ii) EFS or OS; and (iii) time of relapse or death. Relationship between patients' outcome and antigen levels was determined according to the Kaplan-Meier method, considering NB patients with plasma levels of the selected antigen above or below the corresponding cut-off level. EFS and OS curves were compared by the log-rank test using MedCalc software. 

A *P* value 0.05 was considered as statistically significant. Significance ranges are as the following: *P < 0.05; **P < 0.005; and ***P < 0.0005.

## 3. Results

### 3.1. Levels of sHLA-E, sHLA-F, Chromogranin, and Calprotectin Were Increased in NB Patients

First, concentrations of sHLA-E, sHLA-F, chromogranin, and B7H3 were analyzed in plasma samples from 84 NB patients and 75 age-matched healthy children (HC). Concentration of calprotectin was evaluated in serum samples from 33 NB patients and 35 HC.

**Figure 1:**
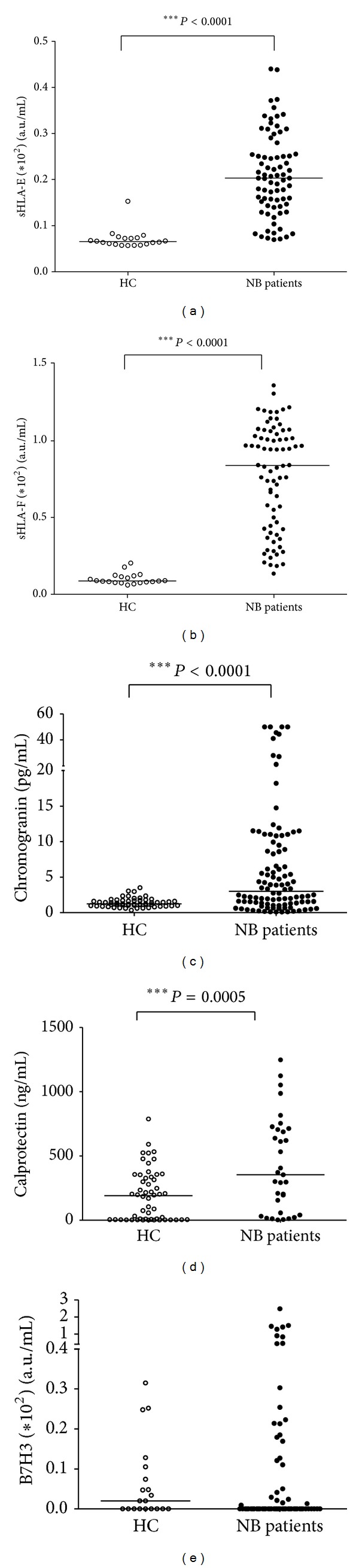
Levels of sHLA-E, sHLA-F, chromogranin, and calprotectin are increased in NB patients. Concentrations of sHLA-E (a), sHLA-F (b), chromogranin (c), calprotectin (d), and B7H3 (e) measured in samples from NB patients (black dots) or healthy children (HC, white dots). Results are expressed as arbitrary units/mL for sHLA-E, sHLA-F, and B7H3, pg/mL for chromogranin and ng/mL for calprotectin. Horizontal bars indicated medians. *P* values are indicated where the difference is statistically significant.

As shown in Figure 1, plasma levels of sHLA-E ((a) arbitrary units/mL ± SD; HC: 0.07 ± 0.02, NB 0.2 ± 0.08, *P* < 0.0001), sHLA-F ((b) arbitrary units/mL ± SD; HC: 0.1 ± 0.03, NB 0.76 ± 0.33, *P* < 0.0001), chromogranin ((c) pg/mL ± SD; HC: 1.4 ± 0.63, NB 6.47 ± 10.41, *P* < 0.0001), and calprotectin ((d) ng/mL ± SD; HC: 3.57 ± 2.76, NB 71.11 ± 120.2, *P* < 0.0001) were significantly higher in NB patients than in HC. Plasma levels of B7H3 ((e) arbitrary units/mL ± SD; HC: 0.064 ± 0.097, NB 0.19 ± 0.45) were not significantly different in NB patients and HC.

### 3.2. Levels of sHLA-E and Chromogranin Were Different between Localized and Metastatic NB Patients

**Figure 2:**
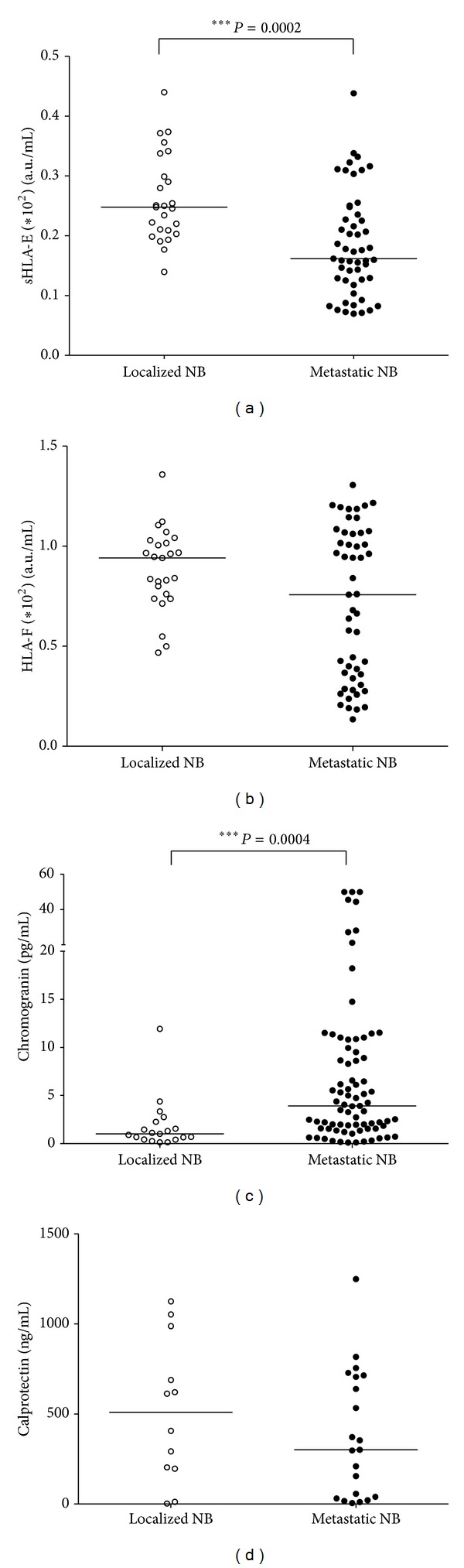
Patients with metastatic NB display higher levels of chromogranin and lower levels of sHLA-E than those with localized NB. Concentrations of sHLA-E (a), sHLA-F (b), chromogranin (c), and calprotectin (d) were analyzed in samples from patients with localized (white dots) or metastatic NB (black dots). Results are expressed as arbitrary units/mL for sHLA-E and sHLA-F, pg/mL for chromogranin and ng/mL for calprotectin. Horizontal bars indicated medians. *P* values are indicated where the difference is statistically significant.

We asked whether concentrations of molecules significantly increased in NB patients were also different between localized and metastatic NB. As shown in Figure 2, plasma levels of sHLA-E were higher in patients with localized than in those with metastatic disease ((a) arbitrary units/mL ± SD; localized: 0.26 ± 0.07, metastatic 0.18 ± 0.08, *P* = 0.0002), whereas plasma levels of sHLA-F ((b) arbitrary units/mL ± SD; localized: 0.88 ± 0.2, metastatic 0.71 ± 0.37) were similar in the two subsets. Plasma levels of chromogranin were higher in patients with metastatic than in those with localized tumors ((c) pg/mL ± SD; localized: 1.86 ± 2.69, metastatic 8.2 ± 11.99, *P* = 0.0004). Finally, serum calprotectin levels were similar in the two patient subsets ((d) ng/mL ± SD; localized: 516.2 ± 394.5, metastatic 381 ± 352.8).

### 3.3. sHLA-E Levels Correlated with sHLA-F Levels

**Figure 3:**
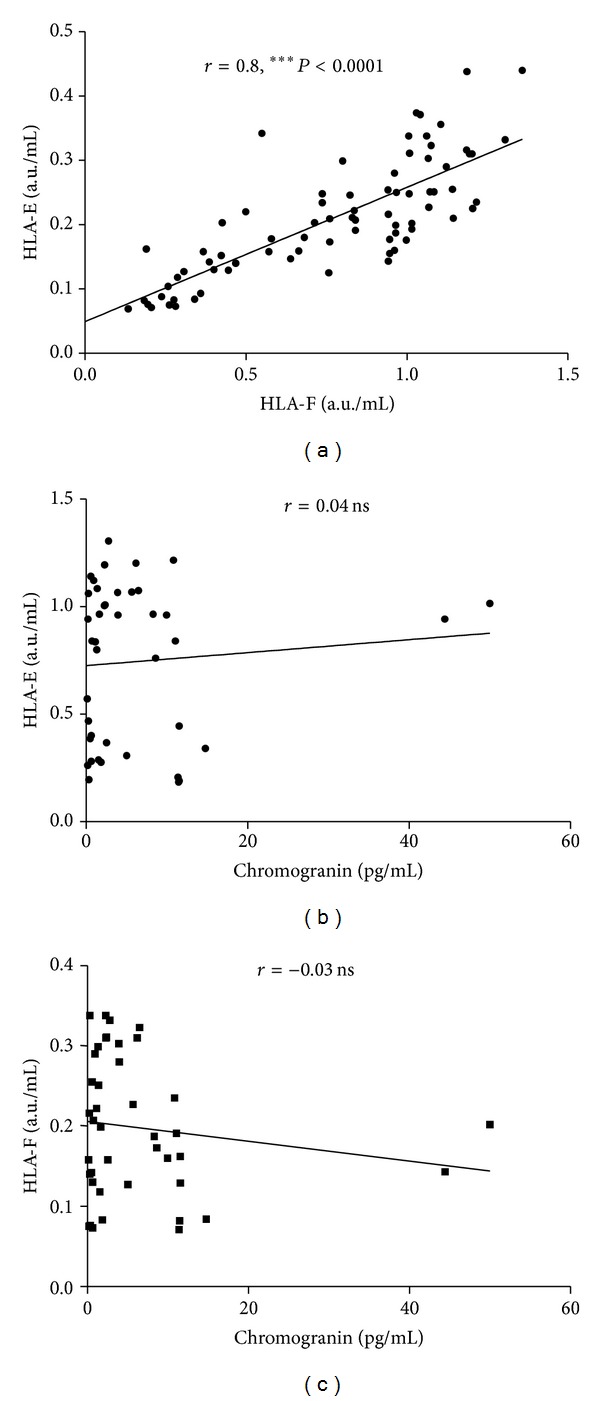
Correlations between plasma levels of sHLA-E, sHLA-F, and chromogranin. Correlation between levels of sHLA-E and sHLA-F (a), chromogranin and sHLA-E (b), and chromogranin and sHLA-F (c). Each point represents single observations. Linear regression and Spearman *r* values are shown. *P* values are indicated when correlation is significant.

We then tested whether plasma levels of sHLA-E, sHLA-F, and chromogranin correlated with each other. As shown in Figure 3(a), levels of sHLA-E and sHLA-F strongly correlated with each other (Spearman = 0.8; *P* < 0.0001), whereas no correlation was found between plasma chromogranin levels and sHLA-E (Figure 3(b), Spearman *r* = 0.04) or sHLA-F levels (Figure 3(c), Spearman *r* = −0.03). 

### 3.4. Levels of Chromogranin, sHLA-E, and sHLA-F at Diagnosis Predicted Outcome of NB Patients

To evaluate whether plasma concentrations of chromogranin, sHLA-E, and sHLA-F at diagnosis may predict clinical outcome of NB patients, we calculated the cut-off levels by ROC curves analysis, using EFS or OS as read-out. 

**Figure 4:**
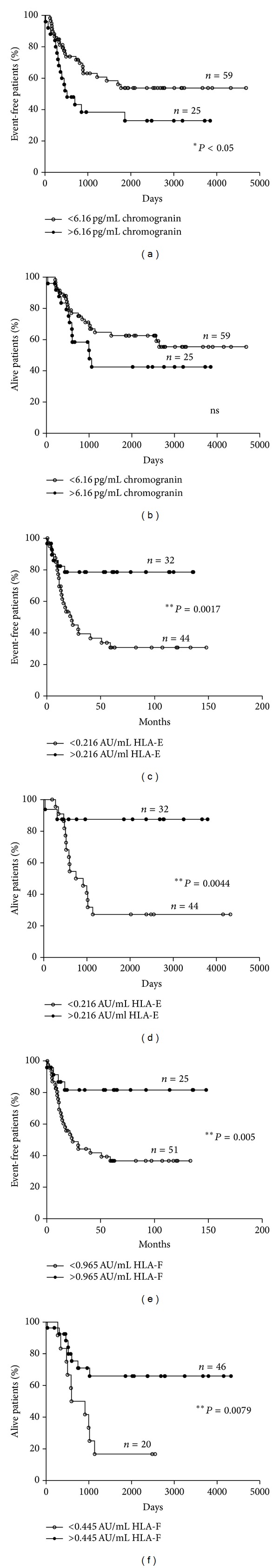
Kaplan-Meier plots of EFS and OS of NB patients stratified by levels of soluble biomarkers. EFS and OS of NB patients stratified according to levels of chromogranin ((a) and (b)), sHLA-E ((c) and (d)), and sHLA-F ((e) and (f)) above (black dots) or below (white dots) the ROC cut-off levels. *Y*-axes indicate proportion of event-free or alive NB patients, whereas *X*-axes indicate survival time (days or months). *P* values are indicated where the difference is significant.

Correlations between chromogranin levels and clinical outcome were analyzed on 74 NB patients with available follow-up data. The cut-off level for chromogranin was 6.16 pg/mL for both OS and EFS. The number of relapse-free patients was higher in patients with chromogranin levels below cut-off (35/59, 59.3%) than that in patients with levels above cut-off (9/25, 36.0%, *P* = 0.03). Conversely, the number of alive patients was similar in patients with chromogranin levels below or above cut-off (37/59 *versus* 12/25, 62.7% versus 48.0%). As shown in Figure 4, Kaplan-Meier analysis confirmed that chromogranin levels below 6.16 pg/mL correlated with a better EFS ((a), *P* < 0.05). However, no correlation was found with OS (Figure 4(b), ns).

Correlations between sHLA-E and sHLA-F levels and clinical data were analyzed on 76 NB patients with sufficient follow-up data. The cut-off level for sHLA-E was 0.216 arbitrary units/mL for both EFS and OS. The number of relapse-free patients was higher in the group of patients with sHLA-E levels above cut-off (25/32, 78.1%) than that in patients with levels below cut-off (17/44, 38.1%, *P* = 0.001). Moreover, the frequency of alive patients was higher in the group of patients with sHLA-E levels above cut-off (28/32, 87.5%) than that in patients with levels below cut-off (16/44, 36.4%, *P* = 0.0008). As shown in Figure 4, Kaplan-Meier analysis confirmed that sHLA-E levels above 0.216 arbitrary units/mL correlated with a better EFS ((c), *P* = 0.0017) and OS ((d), *P* = 0.0044).

Similar data were obtained for sHLA-F. The cut-off level for sHLA-F was 0.965 arbitrary units/mL for EFS and 0.445 arbitrary units/mL for OS. The frequency of relapse-free patients was higher in patients with sHLA-F levels above cut-off (20/25, 80.0%) than that in patients below cut-off (21/51, 41.2%, *P* = 0.02). Moreover, the number of alive patients was higher in patients with sHLA-F levels above cut-off (40/46, 86.9%) than that in patients with levels below cut-off (4/20, 20.0%, *P* = 0.0042). As shown in Figure 4, Kaplan-Meier analysis confirmed that sHLA-F levels above 0.965 arbitrary units/mL correlated with a better EFS ((e), *P* = 0.005) and sHLA-F levels above 0.445 arbitrary units/mL correlated with a better OS ((f), *P* = 0.0079).

**Figure 5:**
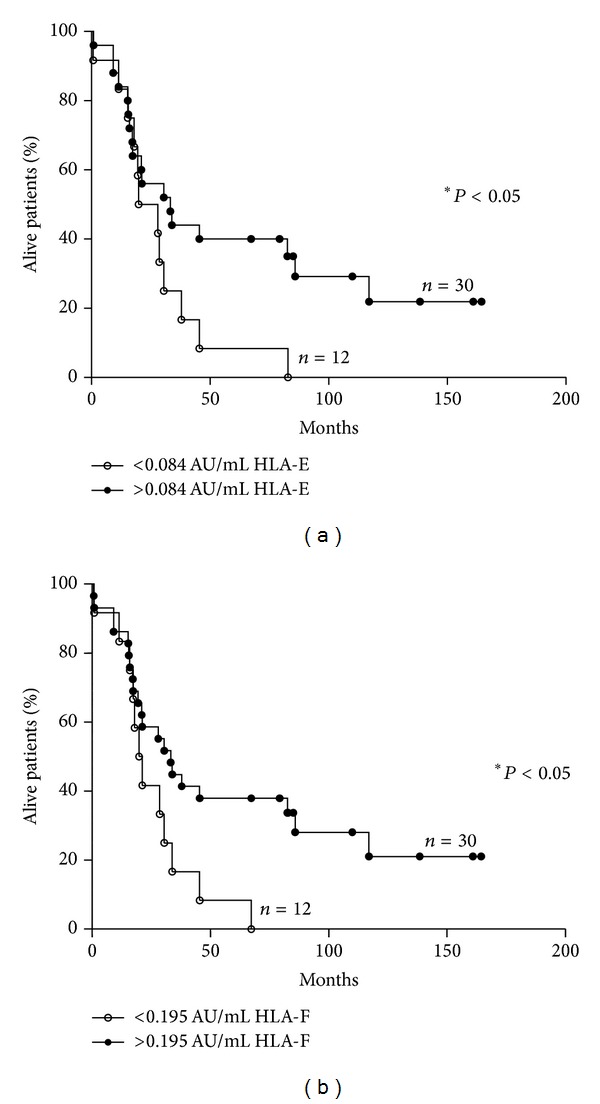
Kaplan-Meier plots of OS of patients with metastatic NB stratified by levels of sHLA-E and sHLA-F. OS of patients with metastatic NB stratified according to levels of sHLA-E (a) and sHLA-F (b) above (black dots) or below (white dots) the ROC cut-off levels. *Y*-axes indicate proportion of alive NB patients, whereas *X*-axes indicate survival time (months). *P* values are indicated where the difference is significant.

Next, the same analysis was performed considering only the 42 patients with metastatic NB. The cut-off level for HLA-E was 0.084 arbitrary units/mL for OS and 0.104 arbitrary units/mL for EFS. The number of alive patients was higher in patients with sHLA-E levels above cut-off (9/30, 30%) than that in patients with levels below cut-off (0/12, 0%, *P* = 0.04). Conversely, the number of relapse-free patients was similar in patients with sHLA-E levels above (14/31, 45.2%) or below (5/11, 45.4%) cut-off level. As shown in Figure 5, Kaplan-Meier analysis confirmed that sHLA-E levels above 0.084 arbitrary units/mL correlated with a better OS ((a), *P* = 0.05) also in this subset of patients. 

The cut-off level for sHLA-F was 0.195 arbitrary units/mL for OS and 0.276 arbitrary units/mL for EFS. The number of alive patients was higher in patients with sHLA-F levels above cut-off (8/30, 26.6%) than that in patients with levels below cut-off (0/12, 0%, *P* = 0.04). Conversely, the number of relapse-free patients was similar in patients with sHLA-F levels above (4/12, 33.3%) or below (9/30, 30%) cut-off level. As shown in Figure 5, Kaplan-Meier analysis confirmed that in patients with metastatic NB sHLA-F levels above 0.195 arbitrary units/mL correlated with a better OS ((b), *P* = 0.05).

### 3.5. Correlations between Chromogranin, sHLA-E, and sHLA-F Levels with Other Prognostic Factors

We next asked whether chromogranin, sHLA-E, and sHLA-F cut-off levels were related to other predictive clinical parameters, such as sex, age at diagnosis, *MYCN* amplification, and stage of the disease. Data are summarized in Table 2.

Chromogranin levels correlated with sex (no. of female patients: below 6.16 pg/mL chromogranin 18/57; above 6.16 pg/mL chromogranin 17/27; *P* = 0.003) but not with other parameters. Conversely, sHLA-E levels correlated with age at diagnosis (no. of patients <18 months: below 0.216 AU/mL 17/47, 31.9%; above 0.216 AU/mL 24/37, 64.8%; *P* = 0.01) and stage of the disease (no. of metastatic patients: below 0.216 AU/mL 35/47, 74.4%; above 0.216 AU/mL 2/37, 5.4%; *P* < 0.0001). For sHLA-F, statistical analysis was performed using the two different cut-off levels calculated for EFS and OS. In both analyses, sHLA-F levels correlated with age at diagnosis (no. of patients <18 months: below 0.445 AU/mL 7/24, 29.1%; above 0.445 AU/mL 34/60, 56.6%; *P* = 0.01; no. of patients <18 months: below 0.995 AU/mL 20/51, 39.2%; above 0.995 AU/mL 21/33, 63.6%; *P* = 0.03) and stage of the disease (no. of metastatic patients: below 0.445 AU/mL 22/24, 91.6%; above 0.445 AU/mL 15/60, 25%; *P* < 0.0001; no. of metastatic patients: below 0.995 AU/mL 33/51, 64.7%; above 0.995 AU/mL HLA-E 4/33, 12.1%; *P* < 0.0001).

## 4. Discussion

In the present study, we demonstrated for the first time that plasma levels of sHLA-E, sHLA-F, and chromogranin and serum levels of calprotectin are increased in NB patients as compared to healthy subjects. Most importantly, we showed that plasma levels of sHLA-E and sHLA-F significantly associated not only with EFS, as occurring for chromogranin levels, but also with OS. Low levels of both sHLA-E and sHLA-F significantly associated with a worse prognosis not only in the whole cohort of patients but also in the subset of patients with metastatic NB. 

sHLA-E is released by different immune and nonimmune cells upon activation [19, 20]. Recently, we demonstrated [18] an increased sHLA-E concentration in cerebrospinal fluid from patients with multiple sclerosis, as compared to patients with other neurological diseases. Moreover, the high intrathechal levels of sHLA-E associated with a better prognosis, suggesting a role of this molecule in the resolution of inflammation. Recently, Allard et al. [21] have shown that melanoma cells released sHLA-E in response to proinflammatory cytokines, suggesting that sHLA-E could be a marker of antitumor immune response. From our study, we cannot conclude whether sHLA-E is released by the neuroblastoma or the immune cells. However, higher concentrations of sHLA-E correlated with a better EFS and OS of NB patients and associated with age <18 months at diagnosis and with localized tumors. Thus, sHLA-E appeared to associate with a stronger anti-NB response that according to Gowda and coworkers [22] would be more effective in young patients with localized tumors. 

To our knowledge, this is the first demonstration in which sHLA-F is present in biological samples. Lee et al. have shown that this molecule is expressed on the surface of lymphocytes upon activation [23]. Since HLA-Ib molecules are generally released as soluble molecules through shedding mediated by metalloproteases [24], it can be envisaged that the presence of plasma sHLA-F may be related to the immune system activation. This hypothesis is supported by the findings that NB patients with high sHLA-F levels display a better EFS and OS than those with low levels and that sHLA-F levels significantly associated with age at diagnosis and disease stage as sHLA-E level. 

Cox regression analysis confirmed that sHLA-E and sHLA-F values were not independent (not shown). Moreover, since these values strongly correlated with each other, the simultaneous analysis of the two markers did not give additional information compared to the separate analysis (not shown).

We also found for the first time that plasma chromogranin levels associated with a worse EFS of NB patients and that its concentration was higher in patients with metastatic than in those with localized NB. Chromogranin is a hydrophilic glycoprotein normally released by neuroendocrine cells that, upon proteolysis, produce bioactive peptides with different paracrine, autocrine, and endocrine functions [25]. In the last years, this molecule has been characterized as a promising biomarker for the diagnosis and monitoring of treatment response of gastroenteropancreatic neuroblastic tumors [10]. Thus, our findings suggest that chromogranin could be a useful biomarker for monitoring treatment response also in NB patients. Future prospective studies will address this possibility. 

Conversely, data obtained on calprotectin, a calcium-binding heterodimeric complex formed by S100A8 and S100A9 proteins, failed to demonstrate its potential use as NB biomarker. Calprotectin serum levels were significantly higher in NB patients than in healthy children; as expected by our demonstration, bone marrow-infiltrating neuroblastoma cells always express this protein [9]. However, calprotectin levels were not significantly different between patients with localized and metastatic tumors, likely because it is physiologically released by the myeloid cells [26]. 

Finally, plasma levels of B7H3, a member of the costimulatory B7 family, with both immune-suppressive and stimulatory functions, expressed on the surface of bone marrow-infiltrating NB cells [9, 27] and primary NB tumors [8], were not significantly different between NB patients and healthy children, thus excluding its potential use as a surrogate biomarker.

In conclusion, we have here identified two novel plasma biomarkers, sHLA-E and sHLA-F, that are able to predict at diagnosis both EFS and OS of NB patients. Future prospective studies will address whether these surrogate biomarkers could be helpful in stratification of high-risk patients into different therapeutic regimens.

## Figures and Tables

**Table 1:** Characteristics and clinical features of NB patients.

Diagnosis	
Gender	
M	49/84
F	35/84
Age	
<18 months	42/84
>18 months	42/84
*MYCN *	
Normal	57/84
Amplified	27/84
Stage	
1	7/84
2	8/84
3	22/84
4	42/84
4s	5/84
Followup	
Overall survival	
CR	41/84
Alive with disease	8/84
Dead	35/84
Relapse-free survival	
Relapsed	40/84
Local	7/84
Disseminated	27/84
Both	6/84

**Table 2:** Correlations between serum biomarkers and clinical parameters.

	Sex		N-myc amplification		Age at diagnosis		Stage	
	F	M	No	Yes	<18 m	>18 m	1–3	4
Chromogranin								
Below 6.16 pg/mL	18	39	40	18	30	28	33	24
Above 6.16 pg/mL	17^a^	10	17	9	11	15	9	18
HLA-E								
Below 0.216 AU/mL	20	23	31	10	17	30	12	35
Above 0.216 AU/mL	15	26	33	10	24^a^	13	35^a^	2
HLA-F								
Below 0.995 AU/mL	14	34	40	14	20	31	18	33
Above 0.995 AU/mL	14	22	18	12	21^a^	12	29^a^	4
Below 0.445 AU/mL	10	14	17	6	7	17	2	22
Above 0.445 AU/mL	22	38	41	20	34^a^	26	45^a^	15

^a^ *P* < 0.05 (Fisher's exact test).
